# Family Structure and History of Childhood Trauma: Associations With Risk-Taking Behavior Among Adolescents in Swaziland

**DOI:** 10.3389/fpubh.2020.563325

**Published:** 2020-10-14

**Authors:** Mokoena Patronella Maepa, Thobile Ntshalintshali

**Affiliations:** ^1^Department of Clinical Psychology, School of Medicine, Sefako Makgatho Health Sciences University, Pretoria, South Africa; ^2^Psychosocial and Behavioural Sciences, Faculty of Health Sciences, North-West University, Potchefstroom, South Africa

**Keywords:** family structure, childhood trauma, risk taking behavior, self-harm behavior, adolescents

## Abstract

**Introduction:** Risk-taking and self-harm behavior among adolescent are a global challenge. This study explored family structure and history of childhood trauma and their association with risk-taking and self-harm behaviors among adolescents in Swaziland.

**Methods:** Using a cross-sectional design, a sample of 470 male and female adolescents were sampled through simple random sampling from selected high schools in Swaziland. They completed a questionnaire assessing family structure, history of childhood trauma, and risk-taking and self-harm behaviors. Analysis of variance and *t*-test were used to analyze the results.

**Results:** The findings revealed that family structure significantly influence risk-taking and self-harm behavior among adolescents [*F*_(2, 247)_ = 5.481; *P* < 0.004] those from child-headed and single-parent households reported higher risk-taking and self-harm behaviors. The results also revealed adolescents history of childhood trauma to be more risk-takers than those without history of childhood trauma *t*_(468)_ = 3.409, *p* < 0.001.

**Discussion:** Study results suggest that family structure and history of childhood trauma have significant association with adolescents' risk-taking and self-harm behaviors.

## Introduction

Globally, the involvement of adolescents in risk-taking behavior has reached an alarming level (1). In Sub-Sahara Africa where more than eight out of 10 of the world's HIV-infected adolescents live, the issue of risk-taking is worse (2). Tull et al. (3) define risk-taking behavior as the “tendency to engage in behaviors that have the potential to be harmful or dangerous, yet at the same time provide the opportunity for some kind of outcome that can be perceived as positive.” The major problematic risky behaviors among young people either in and out of school has been reported to include tobacco, alcohol, and illicit drug use, risky sexual behavior, and self-harm (4). There have also been increasing reports of high school student's engagement in non-suicidal self-injury (NSSI), which has exacerbated the youth's suicide incident (5).

Based on vulnerability to risk-taking, adolescents, who are best defined as young people aged 10–19 years, have been labeled as the most susceptible to the adoption of risky behaviors (6). This is the case because this challenging developmental period is marked by increased levels of curiosity and self-doubt which heightens the potential for engaging in risk-related activities (7). Additionally, significant physical, cognitive, and psychological changes as well as sexual development occurs (8) which prompts sexual experimentation (9). With the developing young men, sexual forces are awakened which fuels the likelihood of engagement in risky sexual behavior (10). Adolescent girls experience the appearance of the first menstruation, their bodies become mature and fruitful and the sexual urge begins and intensifies (8). Furthermore, adolescent's cognitive ability is marked by concrete thinking wherein long term implications of actions are not perceived (11). Adolescents also have elevated levels of egocentrism, which refers to a state of heightened self-consciousness as well as elevated levels of attention-seeking behavior as they attempt to be noticed or visible and consider themselves unique which is all linked to elevated risk-taking behavior like drug use and suicides (8). Psychologically, adolescence is the period of identity formation, integration, and commitment whereby adolescents conform more to peer pressure in their quest to uncover who they are outside their parents, thus making them more prone to risk adoption (9).

The subject of risk-taking behavior among the youth of Swaziland where the study was conducted bears no differences in relation to global trends. The United Nations Population Fund- Swaziland (UNPF) (11, 12) reported that almost 30 and 20% of out of school and in school youth, respectively, reported to be taking alcohol (66.7%), had engaged in sexual intercourse coupled with low levels of condom use at first sex and 45% reported early childbearing experiences (19 years of age) (14) increasing teenage pregnancy.

The high HIV mortality rates which take the parents who are the most productive members of society thus leaving many adolescents to grow up in child-headed families, prone to exploitation, sexual abuse, poverty, and unwanted pregnancies (13) greatly contribute to this phenomenon. These high HIV mortality rates in Swaziland disintegrate the family structure and takes away the opportunity of growing in the traditional two-parented family for many young people, which allegedly possess qualities such as parental warmth and guidance which act as a buffer to risk-adoption (14) such as self-harm behavior (15), sex work (16), and substance abuse.

Alcohol and other drugs (AOD) use among adolescents remains one of this age cohort prominent risk-taking behavior which has been associated with social issues like crime, including sexual and grievous bodily harm, assaults and murder (17), gang activities, vandalism, bullying, and truancy within school premises (18). Additionally, alcohol is alleged to increases the risk of engagement in sexual risk behavior, leading to sexually transmitted infections (STIs) (19) and unplanned pregnancy which in most cases lead to unsafe abortions which are the leading cause of death for women aged 15–19 worldwide (20). There is also a link between substance use and self-harm behavior in adolescents with female adolescents being three times more likely and male adolescents being 17 times more likely to attempt self-harm while under the influence of alcohol (21).

It is against this background that the researchers undertook the study to explore the association of family structure, history of childhood trauma, and risk-taking behaviors among Swaziland adolescents. To our knowledge, no other study on the related influential nature of the aforementioned psychosocial factors on risk-taking behaviors has been conducted in Swaziland, thus this study aims to bridge this gap in knowledge and effect intervention programs.

## Materials and Methods

### Participants

The study was conducted in Manzini region, Swaziland using a cross-sectional research design. Four hundred and seventy (*n* = *470)* male (49.4%) and female (50.6%) youth with ages ranging from 12 to 25 years (mean age was 16.57 years, SD 2.19 years) completed the questionnaires (See detailed sample characteristics on Table 1). Manzini is one of the biggest and densely populated region in Swaziland. Manzini is also the top region by high schools in Swaziland, with a population of about 55 high schools which account for a greater proportion of Swaziland's high schools. Swaziland's total high schools are estimated to be around 398 (22). As such the first approach was to use cluster sampling to divide the schools in to more manageable units. Secondly, purposive sampling was used to select the schools intended for the study, namely a mixed school, a girl's and boy's only school, and a rehabilitation school. Lastly, simple random sampling was thereafter used to select the final sample within the selected schools of study.

**Table 1:** Demographics, family structure, and history of childhood trauma of the adolescents (*N* = 470).

**Age**	
Mean age	16.54
Age range	12–25 years
**Gender**	***n*** **(%)**
Boys	232 (49.4)
Girls	238 (50.6)
**Level of education**	
Form 1	93 (19.8)
Form 2	94 (20.0)
Form 3	95 (20.2)
Form 4	94 (20.0)
Form 5	94 (20.0)
**No. of biological parents alive**	
None	25 (5.3)
One alive	127 (27.0)
Both alive	318 (67.7)
**Biological parents living with**	
None	52 (11.1)
One parent	210 (44.7)
Both parents	208 (44.3)
**History of childhood trauma**	
Childhood trauma	52 (11.1)
No childhood trauma	418 (88.9)

### Procedure

Permission to conduct the study was firstly obtained from the ethics committee of the North-West University (NWU-HS-2017-0200). Further permission was obtained from the Ministry of education in Swaziland and from the principals of the different participatory schools, which also included permission from the subject teachers whose time will be utilized for data collection.

Learners were provided with the study information by the researcher and their teachers. They were given letters that contained the study information and requests for permission to give to their parents or guardians. In the letters parents or guardians were asked to sign consent forms indicating that they allow their children to take part in this study. Only upon receipt of signed consent forms from parents, learners were requested to sign assent forms. The information that was provided to the learners before signing the assent forms included indicating to the participants that participation is on voluntary bases and they are free to withdraw from the study at any given time if they see the need to do so. Participants were assured that a high degree of privacy and confidentiality will be maintained and participation is anonymous.

The participants were given a brief guideline on how the questionnaires are to be completed. The questionnaires included written instructions on how questions are to be answered and that there is no right or wrong answer. Questionnaires were completed by the learners in the presence of the researcher for any clarification purposes during the designated times. These questionnaires were in paper and pencil format. Participants completed these questionnaires during the free period, not interrupting with class lessons.

## Measures

### Biographic Information

A questionnaire has a section with demographic information of participants such as their age, gender, race, location of the school, and grade.

### Family Structure

Family structure is defined as a group consisting of parents and their children or any other person related by blood or marriage (23). Family structure was measured through assessment questions inquiring about the number of biological parents one has or is living with (both/one/none as informed by literature.

### Childhood Trauma Questionnaire (CTQ)

Childhood trauma is defined as experiences of abuse (physical, emotional, or sexual abuse), neglect, and household dysfunction of varying frequency, severity, and duration before the age of 18 (23). Childhood trauma was measured using the Childhood Trauma Questionnaire (CTQ) which is a 28-item self-report measure designed to assess five types of negative childhood experiences: (1) emotional neglect, (2) emotional abuse, (3) physical neglect, (4) physical abuse, and (5) sexual abuse (24). Three additional items assess tendencies of respondents to minimize or deny abuse experiences. Respondents rate the truth of each statement on a 1–5 scale point Likert scale with responses ranging from 0 (never) to 5 (always). The CTQ has demonstrated reliability and validity, including test-retest reliability coefficients ranging from 0.79 to 0.86 over an average of 4 months, internal consistency reliability coefficients ranging from a median of 0.66 to a median of 0.92 across a range of samples (25). This scale has not been used in Swaziland before, thus a pilot study was first conducted to validate its reliability in the Swazi population. A Cronbach's alpha coefficient of 0.77 was obtained for the scale in the pilot study.

### The Risk-Taking and Self-Harm Inventory for Adolescents Measure (RTSHIA)

Risk-taking behavior (RTB) refers to the tendency to engage in behavior that has the potential to be harmful or dangerous (26). The RTSHIA was adopted to assess risk-taking behavior among adolescents in Swaziland. This is a self-report measure designed to assess adolescent risk-taking (RT) and self-harm (SH) (27). The scale consists of 38 items which are rated on a four-point Likert scale from 0 = *Never* if the statement does not apply to one to 3 = *Many times* if so. The RTSHIA has high reliability for both components with Cronbach's alphas ranging 0.85 and 0.93 & 87 and 0.90 (28). This scale has not been used in Swaziland before, and as a result, a pilot study for this current study with a smaller sample size yielding a Cronbach's alpha coefficient of 0.81 for the risk-taking sub- scale and a Cronbach's alpha of 0.84 for the self-harm subscale was conducted before the main study.

### Statistical Analysis

The IBM Statistical Package for the Social Sciences *(SPSS 24)* was used to analyze the data. The *one-way Analysis of variance (ANOVA)* was used to examine if there were any observed associations in risk-taking behavior between adolescents from single-parent or child-headed households from those from two-parent households. Additionally, a *post-hoc test* was used for additional exploration of the differences between group means.

The *Independent samples t-test* was also used to examine if there were significant differences between adolescents with a history of childhood trauma and those without history of childhood trauma concerning risk-taking behavior. All tests held a statistical significance at *p* < 0.05.

## Results

### Sample Characteristics

#### Family Structure and Risk-Taking Behavior

The type of family structure significantly influence risk-taking behavior among adolescents [*F*_(2, 247)_= 5.481; *p* < 0.004]. Adolescents from child-headed and single-parent families reported higher risk-taking and self-harm behaviors. Subsequently, there was a need to compute a *post-hoc* multiple comparisons since there was an interaction influence of the type of family on risk-taking behavior of adolescents.

The *post-hoc* result in Table 2 showed that adolescents from child-headed families (M = 14.1458, SD 8) significantly reported higher risk-taking behavior than those from single-parent family (M = 11.28, SD = 7.8) and those from two-parent family (M = 9.89, SD = 8.5).

**Table 2:** *post-hoc test* showing significance of type od family on risk-taking behavior of adolescents.

**Type of family**	**1**	**2**	**3**	***N***	**M (SD)**
1. Child-headed family	–			48	14.14 (8.48)
2. Single-parented family	2.682*	–		215	11.28 (7.84)
3. Two-parented family	4.247*	1.385	–	207	9.89 (8.55)

Significance level **p < 0.001.

#### Childhood Trauma and Risk-Taking Behavior

The result in Table 3 shows there is a significant statistical difference between adolescents with history of childhood adversity and those without history of childhood trauma concerning risk-taking behavior *t*_(468)_ = 3.409, *p* < 0.001. Adolescents with a history of childhood adversity (M = 12.3915, SD = 8.0445) significantly reported higher risk-taking behaviors than those without a history of childhood trauma (M = 9.7946, SD = 8.3583).

**Table 3:** Independent sample *t*-test showing the difference of history of childhood trauma on risk-taking behavior.

**History of trauma**	***N***	**M (SD)**	**DF**	***t***	***P***
With history	212	12.39 (8.04)	486	3.409	0.001
Without history	258	9.79 (8.35)			

Significance level **p < 0.001.

## Discussion

This study aimed to explore the association of family structure and history of childhood traumaconcerning risk-taking behavior among a sample of 470 adolescents in Swaziland. The study revealed that adolescents from child-headed families, followed by those from single-parented families engaged in higher risk-taking behavior than those from two-parented families. These results coincide with past literature. For example, Kheswa and Tikimana (29) concluded that due to the death of parental figures mostly as a result of the HIV/AIDS epidemic, many adolescents are left in child-headed households and tend to engage in risk-taking behavior such as unhealthy sexual practices and alcohol abuse. Additionally, in Swaziland, it has been uncovered that one of the underlying factors promoting alcohol and substance usage is the lack of parental guidance due to the high HIV mortality rates leaving most children parentless thus more prone to risk adoption (9). This can be explained by the fact that parents are said to serve as a source of external monitoring (parental behaviors involving attention to and tracking of children's whereabouts and activities) throughout childhood and adolescence (30). In the early years, parental monitoring is necessary as a source of protection for children (31). But even as children mature, high levels of parental monitoring remain an important factor that predicts adolescent health behaviors such as drug use, as well as other behavioral problems (31).

Family members (typically parents) are said to form the foundation for close, important relationships throughout childhood and adolescence, which act as a protective barrier against risk adoption (32). However, the pattern of interaction is such that an association between lack of protective resources like parental figures and poorer functioning (e.g., higher levels of substance use) is most clearly evident for youth with relatively low assessed exposure to adversity about risk (33). However, a study by Foster (34) defies this finding as they uncovered that most children from child-headed families in Africa are still being cared for by members of their extended family, called the traditional safety net for orphans, which serve as a protective barrier against vulnerability to risk adoption.

Additionally, this study uncovered that a greater percentage of the participants live in single-parented households, mostly with their biological mothers, and was the second-largest group prone to risk adoption. Bird et al. (35) concurs with these findings as it was found that Mexican adolescents from single-parented families (predominantly led by mothers) were 2.0 times more likely to be current smokers and experienced less or even careless supervision than those from two-parented households. Single mothers experience greater parenting stress and have less time and assistance in supervising children, as well as less time to develop and maintain the supportive bonds that expressively control children (36).

However, a study by Zisk et al. (37) views that throughout childhood, adolescence, and even into the college years, parents, primarily mothers, remain the most frequently identified primary attachment, nurturing, and protective figure for youth. Contrary to that finding, other studies view that the absence of an important potential source of guidance, nurturance, and support (being an absent father), can increase the likelihood of both substance use and violence-related behavior among youth (33). This indicates that not having a parental figure regardless of mother or father predisposed youth to risk-taking behavior. Also, “risky families” (families with high conflict, aggression, and cold, unsupportive, neglectful, and absent relationships) are said to being more likely to have children with disruptions in stress-responsive biological systems, poorer health behaviors (30).

The results of the study further indicated that adolescents from two-parent households reported lesser risk-taking behavior compared to other family structures. These findings are validated by the works of previous writers who documented that adolescents from such households reported a delay or reduction of engagement in sexual activity (38); reduced levels of self-harm compared to those from the stepparent, single-parented or no-parent groups (19) and receive higher levels of parental closeness and monitoring, dimensions of a family structure which act as a buffer against the adoption of risk-taking behavior adolescents (39). Moreover, emerging data suggest that during childhood and adolescence, close family relationships can ameliorate the impact that adversity has on lifespan physical and mental health (30).

The study findings indicates that adolescents who reported history of childhood trauma have been engaging in risk-taking behavior. These findings coincide with arguments from criminological theories such as general strain theory (40), which holds that recent adversities are more likely to be associated with maladaptive behavior such as delinquency, substance abuse, or criminal behavior because victims are using these methods to cope with traumatic stress. Furthermore, several past and present studies validate the study results that exposure to adverse childhood experiences (ACE) subsequently leads to the adoption of smoking (41) and early alcohol use among adolescents (42). Also, an alarming rate of suicide attempts (43) and non-suicidal self-harm (NSSH) is attributed to early childhood trauma (44).

The experience of adversity in early life is also found to be associated with increased risk for earlier onset of any physical disease, and any emotional, nervous, or psychiatric disorder especially in later life, which shows the lasting legacy of childhood adversity for not only maladaptive behavior but also disease risk in later life (45). Childhood adversity may also affect the child's ability to connect with school, which is a critical influence in an adolescent's development (46). In turn, failure to bond with the school could increase the risk for deviant behavior and psychological distress (43).

The association between history childhood adversity and risk-taking behavior can be understood as a result of psychosocial negative experiences at both micro-and macro-level, consisting of negative caregiving environment (e.g., childhood maltreatment, caregiver psychopathology, death, and depriving care environment), family context (e.g., familial conflict, domestic violence, addiction, and marital dissolution), community environment (e.g., violence/crime and poor infrastructure), and societal environment (e.g., overt discrimination, gender inequality, and political and economic exclusion) (47). Exposure to such adverse events or environments in childhood is said to be particularly harmful as early childhood is an exceptionally salient period for further development of psychological well-being (48).

## Conclusion

This study shows that family structure and history of childhood trauma play a role in adolescents' engagement in risk-taking and self-harm behavior. Researchers, therefore, recommend that at-risk adolescents, i.e., those who come from child-headed and single-parent families, and those with a history of childhood trauma receive intervention before engaging in risk-taking behavior. Some life skills training programs be included in the school curriculum to help empower leaners in general.

## Strengths and Limitations

The unique sample as it included adolescents from different schools with different population characteristics serves as a strength for this study. The scales of the research were used for the first time in Swaziland and they were found to be valid and reliable which can enable other researchers to use this as a point of reference in future studies.

However, due to the cross-sectional nature of the study, causality cannot be inferred and the findings cannot be generalized to the whole Swaziland. Additionally, given that the participants were sampled using a simple random sample, the researcher lacked available knowledge concerning the population, as such, no control over extraneous factors. The measures were self-report in nature. Additionally, cluster sampling was used to divide the school population into more manageable units or clusters which might have not been an accurate representation of the entire population. Data from the primary care-givers would have been beneficial to further understand the family dynamics.

## Implications for Future Research

The findings of this study indicate that, future studies are necessary to understand the dynamics of risk-taking behavior among adolescents. Future studies can explore the role played by parenting styles, poverty, and exposure to violence in risk-taking behavior.

## Data Availability Statement

The raw data supporting the conclusions of this article will be made available by the authors, without undue reservation.

## Ethics Statement

The studies involving human participants were reviewed and approved by North-West University Ethics Committee. Written informed consent to participate in this study was provided by the participants' legal guardian/next of kin.

## Author Contributions

MM was responsible for the study design, literature review, and writing of the manuscript. TN was responsible for literature review, data collection, and writing of the manuscript. All authors contributed to the article and approved the submitted version.

## Conflict of Interest

The authors declare that the research was conducted in the absence of any commercial or financial relationships that could be construed as a potential conflict of interest.

## References

[1] Loke A Mak Y Wu C. The association of peer pressure and peer affiliation with the health risk behaviors of secondary school students in Hong Kong. Public Health. (2016) 137:113–23. 10.1016/j.puhe.2016.02.024 27062065

[2] Adejumo OA Malee KM Ryscavage P Hunter SJ Taiwo BO. Contemporary issues on the epidemiology and antiretroviral adherence of HIV-infected adolescents in sub-Saharan Africa: a narrative review. J Int AIDS Soc. (2015) 18:1–19. 10.7448/IAS.18.1.20049 26385853 PMC4575412

[3] Tull MT Weiss NH McDermott MJ Post-traumatic stress disorder and impulsive and risky behavior: overview and discussion of potential mechanisms. In: Martin CR Preedy VR Patel VB editors. Comprehensive Guide to Post-Traumatic Stress Disorders. Cham: Springer International Publishing Switzerland (2016). p. 803–16.

[4] MacArthur G Caldwell DM Redmore J Watkins SH Kipping R White J. Individual-, family-, and school-level interventions targeting multiple risk behaviours in young people. Cochrane Database Syst Rev. (2018) 10:CD009927. 10.1002/14651858.CD009927.pub2 30288738 PMC6517301

[5] Tanner A Hasking P Martin G. Co-occurring non-suicidal self-injury and firesetting among at-risk adolescents: experiences of negative life events, mental health problems, substance use, and suicidality. Arch Suicide Res. (2016) 20:233–49. 10.1080/13811118.2015.1008162 26214360

[6] Pirani E Matera C Who is at risk? Gendered psychological correlates in Italian students' sexual risk profiles. Genus. (2020) 76:1–21. 10.1186/s41118-020-00080-9

[7] Magson NR Craven RG Munns G Yeung AS It is risky business: can social capital reduce risk-taking behaviours among disadvantaged youth? J Youth Stud. (2016) 19:569–92. 10.1080/13676261.2015.1098776

[8] Ashok K Unit-1 Introduction to Adolescence. IGNOU (2018). Available online at: http://egyankosh.ac.in//handle/123456789/43368 (accessed April 12, 2020).

[9] Clark DA Donnellan MB Durbin CE Nuttall AK Hicks BM Robins RW. Sex, drugs, and early emerging risk: examining the association between sexual debut and substance use across adolescence. PLoS ONE. (2020) 15:e0228432. 10.1371/journal.pone.0228432 32027682 PMC7004326

[10] Simon W. Sexual Conduct: The Social Sources of Human Sexuality. New York, NY: Routledge (2017).

[11] Smith RJ The relationship between adolescent prosocial moral reasoning, risk taking, and perceived environment to prosocial behaviors (PhD diss.). Fielding Graduate University (2017). Available online at: https://search.proquest.com/openview/ab6db3ac1e8d8a4d4724aade16291468/1?pq-origsite=gscholar&cbl=18750&diss=y (accessed March 12, 2020).

[12] United Nations Population Fund- Swaziland (UNPF) Swaziland: A Total Market Approach for Male Condoms. (2016). Available online at: https://www.unfpa.org/sites/default/files/pubpdf/psi_Swaziland_Dec16final%5Bsmallpdf.com%5D.pdf (accessed April 17, 2020).

[13] Fielding-Miller R Mnisi Z Adams D Baral S Kennedy C. There is hunger in my country: a qualitative study of food security as a cyclical force in sex work in Swaziland. BMC Public Health. (2014) 14:1–10. 10.1186/1471-2458-14-79 24460989 PMC3905928

[14] UNICEF Profile of Adolescents in Swaziland. (2015). Available online at: https://www.unicef.org/about/annualreport/files/Swaziland_2015_COAR.pdf (accessed March 12, 2020).

[15] Lee SY Park EC Han KT Kim SJ Chun SY Park S. The association of level of internet use with suicidal ideation and suicide attempts is South Korean adolescents. A focus on family structure and household economic status. Can J Psychiatry. (2016) 61:243–51. 10.1177/0706743716635550 27254417 PMC4794961

[16] Richardson ET Collins SE Kung T Jones JH Tram KH Boggiano VL. Gender inequality and HIV transmission: a global analysis. J Int AIDS Soc. (2014) 17:e19035. 10.7448/IAS.17.1.19035 24976436 PMC4074603

[17] Fuhr DC Fleischmann A Riley L Kann L Poznyak V Alcohol and other psychoactive substances in Africa and the Americas: results from the WHO Global School-based Student Health Survey. J Substance Use. (2014) 19:274–82. 10.3109/14659891.2013.824038

[18] Manu E Maluleke XT Douglas M Knowledge of high school learners regarding substance use within high school premises in the buffalo flats of east London, Eastern Cape Province, South Africa. J Child Adolesc Substance Abuse. (2017) 26:1–10. 10.1080/1067828X.2016.1175984

[19] Ramsoomar L Risk and protection: alcohol use among urban youth within the birth to twenty (BT20) cohort (PhD thesis). University of Witwatersrand, Johannesburg, South Africa (2016). Available online at: https://pdfs.semanticscholar.org/61d2/f640f1c7e5757c1ea1e7cff7c97263fce5c6.pdf. (accessed March 12, 2020).

[20] Jonas K Crutzen R van den Borne B Sewpaul R Reddy P. Teenage pregnancy rates and associations with other health risk behaviours: a three-wave cross-sectional study among South African school-going adolescents. Reprod Health. (2016) 13:50–60. 10.1186/s12978-016-0170-8 27142105 PMC4855358

[21] Groves SA Stanley BH Sher L. Ethnicity and the relationship between adolescent alcohol use and suicidal behavior. Int J Adolesc Med Health. (2007) 19:19–25. 10.1515/IJAMH.2007.19.1.19 17458320

[22] Lee Y-R Schooling and immunization of orphaned children in Swaziland (PhD diss.). KDI School (2016). Available online at: https://archives.kdischool.ac.kr/bitstream/11125/30583/1/Schooling%20and%20immunization%20of%20orphaned%20children%20in%20Swaziland.pdf (accessed March 11, 2020).

[23] Karatekin C Hill M. Expanding the original definition of adverse childhood experiences (ACEs). J Child Adolesc Trauma. (2019) 12:289–306. 10.1007/s40653-018-0237-5 32318200 PMC7163861

[24] Lee S Family structure effects on student outcomes. In: Schneider S Coleman JS editors. Parents, Their Children, and Schools. New York, NY: Routledge (2018). p. 43–76.

[25] Cecil CAM Viding E Fearon P Glaser D McCrory EJ. Disentangling the mental health impact of childhood abuse and neglect. Child Abuse Neglect. (2017) 63:106–19. 10.1016/j.chiabu.2016.11.024 27914236

[26] Azmawati MN Hazariah ABS Shamsul AZ Norfazilah A Azimatun NA Rozita H Risk taking behaviour among urban and rural adolescents in two selected districts in Malaysia. South African Fam Pract. (2015) 57:160–5. 10.1080/20786190.2014.977048

[27] Vrouva I Fonagy P Fearon P Rossouw T. The risk-taking and self-harm inventory for adolescents: development and psychometric properties. Psychol Assess. (2010) 22:852–65. 10.1037/a0020583 20919771

[28] Xavier A Pinto-Gouveia J da Cunha MA The protective role of self-compassion on risk factors for non-suicidal self-injury in adolescence *School Mental Health*. (2016) 8:476–85. 10.1007/s12310-016-9197-9

[29] Kheswa JG Tikimana S Criminal behaviour, substance abuse and sexual practices of South African adolescent males. J Psychol. (2015) 6:10–8. 10.1080/09764224.2015.11885519

[30] Chen E Brody GH Miller GE. Childhood close family relationships and health. Am Psychol. (2017). 72:555–66. 10.1037/amp0000067 28880102 PMC5598786

[31] Jones JD Cassidy J Shaver PR. Parents' self-reported attachment styles: a review of links with parenting behaviours, emotions, and cognitions. Personal Soc Psychol Rev. (2015). 19:44–76. 10.1177/1088868314541858 25024278 PMC4281491

[32] Smetana JG Campione-Barr N Metzger A. Adolescent development in interpersonal and societal contexts. Annu. Rev. Psychol. (2006) 57:255–84. 10.1146/annurev.psych.57.102904.190124 16318596

[33] Day J Ji P DuBois DL Silverthorn N Flay B Cumulative social-environmental adversity exposure as predictor of psychological distress and risk behaviour in urban youth. Child Adolesc Soc Work J. (2016) 33:219–35. 10.1007/s10560-015-0421-5

[34] Milligan I Withington R Connolly G Gale C Alternative Child Care and Deinstitutionalization in Sub-Saharan Africa: Findings of a Desk Review. Centre For Excellence For Children's Care And Protection (2017). Available online at: https://strathprints.strath.ac.uk/61137/1/Milligan_etal_2016_Alternative_child_care_and_deinstitutionalisation_in_sub_saharan_africa.pdf (accessed July 12, 2020).

[35] Bird Y Staines-Orozco H Moraros J. Adolescents' smoking experiences, family structure, parental smoking and socio-economic status in Ciudad Juárez, Mexico. Int J Equity Health. (2016) 15:1–9. 10.1186/s12939-016-0323-y 26897609 PMC4761169

[36] Osborne C Berger LM Magnuson K. Family structure transitions and changes in maternal resources and well-being. Demography. (2012) 49:23–47. 10.1007/s13524-011-0080-x 22215507 PMC3570825

[37] Zisk A Abbott CH Ewing SK Diamond GS Kobak R. The Suicide Narrative Interview: adolescents' attachment expectancies and symptom severity in a clinical sample. Attachment Hum Dev. (2017) 19:447–62. 10.1080/14616734.2016.1269234 28002988 PMC6103780

[38] Frantz J Sixaba Z Smith M A systematic review of the relationship between family structure and health behaviours amongst young people: an African Perspective. Open Fam Stud. (2015) 7:3–11. 10.2174/1874922401507010003

[39] Stark L Tan TM Muldoon KA King D Lamin DF Lilley S. Family structure and sexual and reproductive health outcomes among adolescents in rural Sierra Leone. Glob Public Health. (2016) 11:309–21. 10.1080/17441692.2015.1031155 25880353

[40] Agnew R Brezina T. General strain theory. In: Krohn MD Hendrix N Hall GP Lizotte AJ editors. Handbook on Crime and Deviance. Cham: Springer (2019). p. 145–60.

[41] Wiehn J Hornberg C Fischer F. How adverse childhood experiences relate to single and multiple health risk behaviours in German public University students: a cross-sectional analysis. BMC Public Health. (2018) 18:1–13. 10.1186/s12889-018-5926-3 30103728 PMC6090638

[42] Ramos-Olazagasti MA Bird HR Canino GJ Duarte CS. Childhood adversity and early initiation of alcohol use in two representative samples of Puerto Rican youth. J Youth Adolesc. (2017) 46:28–44. 10.1007/s10964-016-0575-2 27681408 PMC5639699

[43] Moffitt TE Adolescence-limited and life-course-persistent antisocial behaviour: a developmental taxonomy. In: Biosocial Theories of Crime. New York, NY: Routledge (2017). p. 69–96. 8255953

[44] Han A Wang G Xu G Su P. A self-harm series and its relationship with childhood adversity among adolescents in mainland China: a cross-sectional study. BMC Psychiatry. (2018) 18:28. 10.1186/s12888-018-1607-0 29390995 PMC5796511

[45] McCrory C Dooley C Layte R Kenny RA. The lasting legacy of childhood adversity for disease risk in later life. Health Psychol. (2015) 34:687–96. 10.1037/hea0000147 25150540

[46] Fothergill K Ensminger ME Doherty EE Juon HS Green KM. Pathways from early childhood adversity to later adult drug use and psychological distress: a prospective study of a cohort of African Americans. J Health Soc Behav. (2016) 57:223–39. 10.1177/0022146516646808 27284077 PMC5787376

[47] Berens AE Jensen SK Nelson CA. Biological embedding of childhood adversity: from physiological mechanisms to clinical implications. BMC Med. (2017) 15:135. 10.1186/s12916-017-0895-4 28724431 PMC5518144

[48] Yang L Hu Y Silventoinen K Martikainen P Childhood adversity and depressive symptoms among middle-aged and older Chinese: results from China health and retirement longitudinal study. Aging Mental Health. (2019) 1:923–31. 10.1080/13607863.2019.1569589 30700138

